# 4-Methyl-6-(piperidin-1-yl)pyrimidin-2-amine

**DOI:** 10.1107/S1600536812050982

**Published:** 2013-01-09

**Authors:** S. Sreenivasa, K. E. ManojKumar, T. Srinivasan, P. A. Suchetan, B. S. Palakshamurthy, D. Velumurgan

**Affiliations:** a Department of Studies and Research in Chemistry, Tumkur University, Tumkur, Karnataka 572 103, India; b Centre of Advanced Study in Crystallography and Biophysics, University of Madras Guindy Campus, Chennai 600 025, India; c Department of Studies and Research in Chemistry, U.C.S, Tumkur University, Tumkur, Karnataka 572 103, India; d Department of Studies and Research in Physics, U.C.S., Tumkur University, Tumkur, Karnataka 572 103, India

## Abstract

The title compound, C_10_H_16_N_4_, crystalizes with two mol­ecules (*A* and *B*) in the asymmetric unit in which the dihedral angles between the piperidine and pyrimidine rings are 47.5 (1) and 10.3 (1)°. The four C atoms of the pyrimidine ring in one of the mol­ecules are disordered over two sets of sites with occupancy factors 0.508 (11):0.492 (11). In the crystal, the *A* mol­ecules are linked to one another through N—H⋯N hydrogen bonds, generating *R*_2_^2^(8) ring patterns and forming inversion dimers. These dimers are further connected on either side to a *B* molecule through pairs of N—H⋯N hydrogen bonds, resulting in a tetra­meric unit.

## Related literature

For background to pyrimidine derivatives and their biological activity, see: Patel *et al.* (2003 ▶) and for a related structure see: Sreenivasa *et al.* (2012 ▶). For hydrogen bond motifs, see: Bernstein *et al.* (1995 ▶).
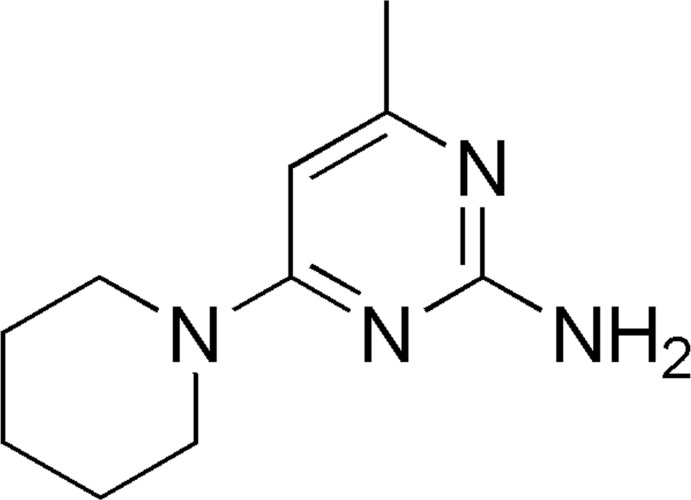


## Experimental

### 

#### Crystal data

C_10_H_16_N_4_
*M* *_r_* = 192.27
Monoclinic, 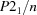

*a* = 13.9605 (4) Å
*b* = 8.7564 (3) Å
*c* = 17.7055 (6) Å
β = 104.381 (2)°
*V* = 2096.57 (12) Å^3^
*Z* = 8
Mo *K*α radiation
μ = 0.08 mm^−1^
*T* = 293 K
0.24 × 0.22 × 0.20 mm

#### Data collection

Bruker APEXII diffractometer
Absorption correction: multi-scan (*SADABS*; Bruker, 2004 ▶) *T*_min_ = 0.972, *T*_max_ = 0.985
15644 measured reflections
3708 independent reflections
2657 reflections with *I* > 2σ(*I*)
*R*_int_ = 0.027

#### Refinement

*R*[*F* ^2^ > 2σ(*F* ^2^)] = 0.051
*wR*(*F* ^2^) = 0.171
*S* = 1.10
3708 reflections
308 parameters
H atoms treated by a mixture of independent and constrained refinement
Δρ_max_ = 0.30 e Å^−3^
Δρ_min_ = −0.20 e Å^−3^

### 

Data collection: *APEX2* (Bruker, 2004 ▶); cell refinement: *APEX2* and *SAINT-Plus* (Bruker, 2004 ▶); data reduction: *SAINT-Plus* and *XPREP* (Bruker, 2004 ▶); program(s) used to solve structure: *SHELXS97* (Sheldrick, 2008 ▶); program(s) used to refine structure: *SHELXL97* (Sheldrick, 2008 ▶); molecular graphics: *ORTEP-3* (Farrugia, 2012 ▶); software used to prepare material for publication: *SHELXL97*.

## Supplementary Material

Crystal structure: contains datablock(s) I, global. DOI: 10.1107/S1600536812050982/sj5288sup1.cif

Structure factors: contains datablock(s) I. DOI: 10.1107/S1600536812050982/sj5288Isup2.hkl

Supplementary material file. DOI: 10.1107/S1600536812050982/sj5288Isup3.cml

Additional supplementary materials:  crystallographic information; 3D view; checkCIF report

## Figures and Tables

**Table 1:** Hydrogen-bond geometry (Å, °)

*D*—H⋯*A*	*D*—H	H⋯*A*	*D*⋯*A*	*D*—H⋯*A*
N3—H*N*3*B*⋯N1^i^	0.86 (2)	2.19 (2)	3.043 (2)	173.3 (19)
N3—H*N*3*A*⋯N5	0.90 (2)	2.34 (2)	3.210 (2)	162.1 (17)

Symmetry code: (i) 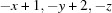.

**Fig. 1.**
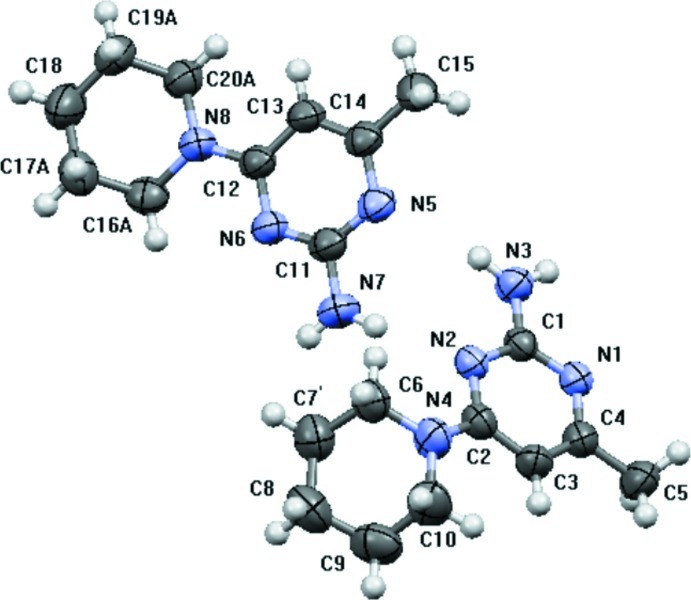
Molecular structure of the title compound, showing the atom numbering scheme. Displacement ellipsoids are drawn at the 50% probability level.

**Fig. 2.**
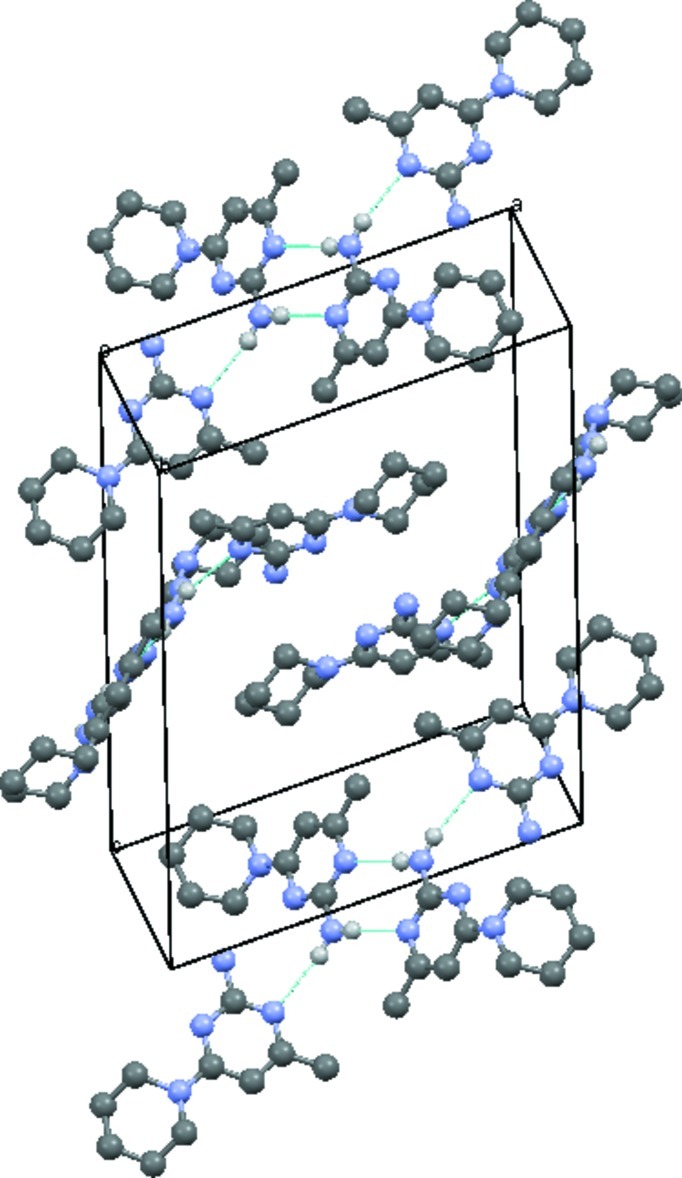
Crystal packing of the title compound with hydrogen bonds drawn as dashed lines.

## supplementary crystallographic information

### Comment

Compounds with a nitrogen-containing heterocyclic ring, such as pyrimidine, are promising candidates for drug design. Pyrimidine derivatives form a component in a number of useful drugs and are associated with many biological and therapeutic activities (Patel *et al.*, 2003). With this in mind, we synthesized the title compound to study its crystal structure.

The title compound crystallizes with two molecules in the asymmetric unit with the piperidine rings in each molecule adopting chair conformations. The dihedral angles between the piperidine ring and pyrimidine rings in the two molecules are 47.5 (1)^o^ and 10.3 (1)^o^ respectively, compared to 14.00 (1)° observed in 1-(2-amino-6-methylpyrimidin-4-yl)-*N*,*N*-dimethylpiperidin-4-aminium chloride (Sreenivasa *et al.*, 2012).

In the crystal structure, the molecules are linked to one another through N—H···N hydrogen bonds generating *R*_2_^2^(8) ring patterns (Bernstein *et al.*, 1995) forming inversion related dimers. These dimers are further connected to one another through a second N—H···N hydrogen bonds resulting in a tetrameric unit.

### Experimental

2-Amino-4-chloro-6-methylpyrimidine(1.39 mmol) was dissolved in acetonirile (3 ml). To this solution, piperidine (1.66 mmol) Xantphos (4,5-bis-diphenylphosphino-9,9-dimethylxanthene), Pd(OAc)_2_ and Cs_2_CO_3_ (0.0695, 0.139 and 2.78 mmole respectively) were added. The reaction mixture was irradiated in a microwave at 60° C for 1.5 hrs. The reaction was monitored by TLC. Acetonitrile was removed under vacuum and the crude product was purified by column chromatography using CH_2_Cl_2_/methanol as eluents. The single-crystal required for the X-ray diffraction was grown by the slow evaporation technique from CH_2_Cl_2_ and MeOH (1:4).

### Refinement

The H atoms bound to carbon were positioned with idealized geometry using a riding model with d(C–H) = 0.93- 0.97 Å. All C–H atoms were refined with isotropic displacement parameters set to 1.2–1.5 U_eq_(C). N—H atoms were located in a difference Fourier map and refined freely. The C16, C17, C19 and C20 carbon atoms of a pyrimidine ring in one of the molecules were disordered over two sites and refined with site occupancy factors 0.508 (11):0.492 (11).

### Crystal data

**Table:**

C_10_H_16_N_4_	Prism
*M**_r_* = 192.27	*D*_x_ = 1.218 Mg m^−^^3^
Monoclinic, *P*2_1_/*n*	Melting point: 455 K
Hall symbol: -P 2yn	Mo *K*α radiation, λ = 0.71073 Å
*a* = 13.9605 (4) Å	Cell parameters from 308 reflections
*b* = 8.7564 (3) Å	θ = 1.7–25.0°
*c* = 17.7055 (6) Å	µ = 0.08 mm^−^^1^
β = 104.381 (2)°	*T* = 293 K
*V* = 2096.57 (12) Å^3^	Prism, colourless
*Z* = 8	0.24 × 0.22 × 0.20 mm
*F*(000) = 832	

### Data collection

**Table:**

Bruker APEXII diffractometer	3708 independent reflections
Radiation source: fine-focus sealed tube	2657 reflections with *I* > 2σ(*I*)
Graphite monochromator	*R*_int_ = 0.027
Detector resolution: 1.20 pixels mm^-1^	θ_max_ = 25.0°, θ_min_ = 1.7°
φ and ω scans	*h* = −16→16
Absorption correction: multi-scan (*SADABS*; Bruker, 2004)	*k* = −10→10
*T*_min_ = 0.972, *T*_max_ = 0.985	*l* = −11→21
15644 measured reflections	

### Refinement

**Table:**

Refinement on *F*^2^	Primary atom site location: structure-invariant direct methods
Least-squares matrix: full	Secondary atom site location: difference Fourier map
*R*[*F*^2^ > 2σ(*F*^2^)] = 0.051	Hydrogen site location: inferred from neighbouring sites
*wR*(*F*^2^) = 0.171	H atoms treated by a mixture of independent and constrained refinement
*S* = 1.10	*w* = 1/[σ^2^(*F*_o_^2^) + (0.0937*P*)^2^ + 0.2814*P*] where *P* = (*F*_o_^2^ + 2*F*_c_^2^)/3
3708 reflections	(Δ/σ)_max_ = 0.006
308 parameters	Δρ_max_ = 0.30 e Å^−^^3^
0 restraints	Δρ_min_ = −0.20 e Å^−^^3^
0 constraints	

### Special details

**Table:**

Geometry. All e.s.d.'s (except the e.s.d. in the dihedral angle between two l.s. planes) are estimated using the full covariance matrix. The cell e.s.d.'s are taken into account individually in the estimation of e.s.d.'s in distances, angles and torsion angles; correlations between e.s.d.'s in cell parameters are only used when they are defined by crystal symmetry. An approximate (isotropic) treatment of cell e.s.d.'s is used for estimating e.s.d.'s involving l.s. planes.
Refinement. Refinement of *F*^2^ against ALL reflections. The weighted *R*-factor *wR* and goodness of fit *S* are based on *F*^2^, conventional *R*-factors *R* are based on *F*, with *F* set to zero for negative *F*^2^. The threshold expression of *F*^2^ > σ(*F*^2^) is used only for calculating *R*-factors(gt) *etc*. and is not relevant to the choice of reflections for refinement. *R*-factors based on *F*^2^ are statistically about twice as large as those based on *F*, and *R*- factors based on ALL data will be even larger.

### Fractional atomic coordinates and isotropic or equivalent isotropic displacement parameters (Å^2^)

**Table:**

	*x*	*y*	*z*	*U*_iso_*/*U*_eq_	Occ. (<1)
C20A	−0.0217 (5)	1.3253 (10)	0.2458 (5)	0.0712 (18)	0.508 (11)
H20A	0.0420	1.3494	0.2804	0.085*	0.508 (11)
H20B	−0.0421	1.4113	0.2110	0.085*	0.508 (11)
C16A	−0.1147 (5)	1.1602 (10)	0.1412 (5)	0.0704 (17)	0.508 (11)
H16A	−0.1335	1.2501	0.1089	0.085*	0.508 (11)
H16B	−0.1116	1.0740	0.1075	0.085*	0.508 (11)
C19A	−0.0960 (6)	1.2997 (13)	0.2928 (5)	0.083 (2)	0.508 (11)
H19A	−0.1003	1.3892	0.3241	0.100*	0.508 (11)
H19B	−0.0767	1.2131	0.3274	0.100*	0.508 (11)
C17A	−0.1892 (5)	1.1300 (10)	0.1878 (5)	0.0796 (19)	0.508 (11)
H17A	−0.1695	1.0418	0.2212	0.096*	0.508 (11)
H17B	−0.2535	1.1095	0.1532	0.096*	0.508 (11)
C20B	−0.0077 (4)	1.2596 (9)	0.2749 (4)	0.0596 (15)	0.492 (11)
H20C	0.0531	1.3173	0.2917	0.072*	0.492 (11)
H20D	−0.0096	1.1832	0.3142	0.072*	0.492 (11)
C16B	−0.1007 (5)	1.0966 (10)	0.1681 (5)	0.0660 (17)	0.492 (11)
H16C	−0.1055	1.0146	0.2038	0.079*	0.492 (11)
H16D	−0.0980	1.0518	0.1185	0.079*	0.492 (11)
C19B	−0.0955 (6)	1.3643 (11)	0.2640 (6)	0.082 (2)	0.492 (11)
H19C	−0.0924	1.4413	0.2253	0.098*	0.492 (11)
H19D	−0.0945	1.4157	0.3127	0.098*	0.492 (11)
C17B	−0.1888 (5)	1.1995 (12)	0.1571 (5)	0.083 (2)	0.492 (11)
H17C	−0.2486	1.1418	0.1352	0.099*	0.492 (11)
H17D	−0.1840	1.2806	0.1210	0.099*	0.492 (11)
N2	0.34290 (11)	0.69695 (17)	0.02892 (9)	0.0568 (4)	
N1	0.45148 (11)	0.81391 (17)	−0.04021 (9)	0.0567 (4)	
N6	0.06632 (13)	1.04169 (19)	0.12363 (10)	0.0651 (5)	
C1	0.39782 (13)	0.8129 (2)	0.01325 (10)	0.0521 (4)	
N5	0.24230 (13)	1.01232 (19)	0.15556 (9)	0.0625 (4)	
N8	−0.01255 (12)	1.1855 (2)	0.19909 (10)	0.0691 (5)	
N3	0.39837 (14)	0.9424 (2)	0.05401 (11)	0.0667 (5)	
C13	0.16605 (15)	1.1656 (2)	0.23497 (11)	0.0633 (5)	
H13	0.1719	1.2282	0.2783	0.076*	
C12	0.07320 (15)	1.1318 (2)	0.18608 (11)	0.0592 (5)	
C3	0.39578 (15)	0.5598 (2)	−0.06970 (13)	0.0666 (5)	
H3	0.3949	0.4715	−0.0991	0.080*	
C2	0.34414 (14)	0.5670 (2)	−0.01117 (12)	0.0595 (5)	
C14	0.24743 (15)	1.1046 (2)	0.21758 (11)	0.0617 (5)	
C4	0.44712 (14)	0.6853 (2)	−0.08232 (11)	0.0602 (5)	
C11	0.15060 (16)	0.9868 (2)	0.11237 (11)	0.0622 (5)	
N4	0.29598 (14)	0.4436 (2)	0.00895 (12)	0.0778 (6)	
C5	0.50160 (19)	0.6890 (3)	−0.14531 (15)	0.0847 (7)	
H5A	0.4642	0.7467	−0.1888	0.127*	
H5B	0.5104	0.5866	−0.1618	0.127*	
H5C	0.5650	0.7360	−0.1257	0.127*	
N7	0.1425 (2)	0.8938 (3)	0.04986 (13)	0.0840 (6)	
C6	0.23128 (17)	0.4557 (3)	0.06164 (14)	0.0758 (6)	
H6A	0.2446	0.5506	0.0906	0.091*	
H6B	0.2446	0.3720	0.0987	0.091*	
C10	0.2798 (2)	0.3038 (3)	−0.03645 (18)	0.0907 (8)	
H10A	0.2916	0.2164	−0.0017	0.109*	
H10B	0.3259	0.2987	−0.0693	0.109*	
C15	0.34854 (17)	1.1346 (3)	0.26812 (13)	0.0859 (7)	
H15A	0.3760	1.0413	0.2928	0.129*	
H15B	0.3444	1.2084	0.3073	0.129*	
H15C	0.3902	1.1734	0.2368	0.129*	
C7	0.12597 (18)	0.4514 (3)	0.01778 (16)	0.0875 (7)	
H7A	0.1102	0.5441	−0.0128	0.105*	
H7B	0.0846	0.4481	0.0544	0.105*	
C18	−0.19444 (19)	1.2695 (4)	0.23650 (17)	0.1021 (9)	
H18A	−0.2134	1.3573	0.2027	0.122*	
H18B	−0.2445	1.2545	0.2652	0.122*	
C9	0.1765 (2)	0.2990 (3)	−0.08581 (17)	0.0976 (9)	
H9A	0.1656	0.2034	−0.1143	0.117*	
H9B	0.1667	0.3816	−0.1234	0.117*	
C8	0.1027 (2)	0.3137 (3)	−0.03636 (18)	0.0964 (8)	
H8A	0.1042	0.2217	−0.0056	0.116*	
H8B	0.0367	0.3243	−0.0700	0.116*	
HN3B	0.4368 (16)	1.015 (3)	0.0472 (12)	0.068 (6)*	
HN3A	0.3653 (15)	0.953 (2)	0.0914 (12)	0.061 (6)*	
HN7B	0.194 (2)	0.851 (3)	0.0398 (15)	0.092 (8)*	
HN7A	0.086 (2)	0.875 (3)	0.0231 (15)	0.086 (9)*	

### Atomic displacement parameters (Å^2^)

**Table:**

	*U*^11^	*U*^22^	*U*^33^	*U*^12^	*U*^13^	*U*^23^
C20A	0.075 (4)	0.070 (4)	0.072 (4)	−0.002 (3)	0.025 (3)	−0.007 (3)
C16A	0.078 (4)	0.074 (4)	0.057 (4)	0.000 (3)	0.011 (3)	0.001 (3)
C19A	0.083 (4)	0.105 (6)	0.068 (4)	−0.003 (4)	0.032 (3)	−0.011 (3)
C17A	0.065 (3)	0.089 (4)	0.084 (4)	−0.008 (3)	0.017 (3)	0.011 (3)
C20B	0.061 (3)	0.059 (4)	0.057 (3)	0.006 (3)	0.012 (2)	−0.007 (2)
C16B	0.057 (3)	0.079 (4)	0.056 (4)	−0.003 (3)	0.000 (3)	−0.002 (3)
C19B	0.086 (4)	0.083 (5)	0.078 (5)	0.020 (4)	0.023 (4)	−0.011 (3)
C17B	0.065 (3)	0.104 (6)	0.073 (4)	0.014 (4)	0.003 (3)	−0.007 (4)
N2	0.0565 (9)	0.0569 (9)	0.0578 (9)	−0.0122 (7)	0.0157 (7)	−0.0061 (7)
N1	0.0557 (9)	0.0573 (9)	0.0586 (9)	−0.0092 (7)	0.0170 (7)	−0.0045 (7)
N6	0.0722 (11)	0.0659 (10)	0.0625 (10)	−0.0134 (8)	0.0269 (8)	−0.0161 (8)
C1	0.0476 (10)	0.0558 (11)	0.0506 (10)	−0.0043 (8)	0.0080 (8)	−0.0007 (8)
N5	0.0732 (11)	0.0646 (10)	0.0541 (9)	−0.0036 (8)	0.0242 (8)	−0.0026 (8)
N8	0.0650 (10)	0.0764 (11)	0.0697 (11)	−0.0104 (8)	0.0240 (8)	−0.0231 (9)
N3	0.0783 (12)	0.0551 (10)	0.0747 (12)	−0.0164 (9)	0.0338 (10)	−0.0126 (9)
C13	0.0738 (13)	0.0672 (12)	0.0518 (11)	−0.0099 (10)	0.0210 (10)	−0.0125 (9)
C12	0.0715 (13)	0.0561 (11)	0.0553 (11)	−0.0114 (9)	0.0257 (10)	−0.0069 (9)
C3	0.0682 (12)	0.0600 (12)	0.0760 (13)	−0.0113 (9)	0.0260 (10)	−0.0174 (10)
C2	0.0543 (10)	0.0578 (11)	0.0660 (12)	−0.0095 (9)	0.0143 (9)	−0.0046 (9)
C14	0.0727 (13)	0.0650 (12)	0.0512 (11)	−0.0045 (10)	0.0228 (10)	0.0006 (9)
C4	0.0548 (11)	0.0660 (12)	0.0611 (11)	−0.0064 (9)	0.0169 (9)	−0.0076 (10)
C11	0.0795 (14)	0.0564 (11)	0.0561 (11)	−0.0109 (10)	0.0272 (11)	−0.0068 (9)
N4	0.0822 (12)	0.0612 (10)	0.0997 (14)	−0.0233 (9)	0.0408 (11)	−0.0137 (10)
C5	0.0928 (17)	0.0897 (16)	0.0841 (16)	−0.0221 (13)	0.0457 (13)	−0.0206 (13)
N7	0.0812 (16)	0.0935 (15)	0.0811 (14)	−0.0078 (12)	0.0272 (12)	−0.0374 (12)
C6	0.0790 (15)	0.0723 (14)	0.0811 (14)	−0.0201 (11)	0.0292 (12)	−0.0006 (12)
C10	0.1000 (19)	0.0553 (13)	0.126 (2)	−0.0135 (12)	0.0452 (17)	−0.0109 (13)
C15	0.0708 (14)	0.120 (2)	0.0663 (14)	−0.0035 (13)	0.0170 (11)	−0.0171 (14)
C7	0.0761 (15)	0.0896 (17)	0.0987 (18)	−0.0061 (13)	0.0251 (13)	0.0031 (15)
C18	0.0704 (16)	0.145 (3)	0.0921 (18)	0.0128 (16)	0.0228 (13)	−0.0197 (18)
C9	0.124 (2)	0.0717 (16)	0.0945 (19)	−0.0238 (15)	0.0227 (17)	−0.0130 (13)
C8	0.0778 (16)	0.0921 (18)	0.111 (2)	−0.0215 (13)	0.0074 (14)	−0.0036 (16)

### Geometric parameters (Å, º)

**Table:**

C20A—C19A	1.500 (11)	N3—HN3B	0.86 (2)
C20A—N8	1.500 (7)	N3—HN3A	0.90 (2)
C20A—H20A	0.9700	C13—C14	1.358 (3)
C20A—H20B	0.9700	C13—C12	1.400 (3)
C16A—C17A	1.504 (11)	C13—H13	0.9300
C16A—N8	1.552 (7)	C3—C4	1.361 (3)
C16A—H16A	0.9700	C3—C2	1.403 (3)
C16A—H16B	0.9700	C3—H3	0.9300
C19A—C18	1.507 (9)	C2—N4	1.366 (2)
C19A—H19A	0.9700	C14—C15	1.495 (3)
C19A—H19B	0.9700	C4—C5	1.498 (3)
C17A—C18	1.507 (7)	C11—N7	1.356 (3)
C17A—H17A	0.9700	N4—C10	1.451 (3)
C17A—H17B	0.9700	N4—C6	1.455 (3)
C20B—N8	1.477 (6)	C5—H5A	0.9600
C20B—C19B	1.504 (11)	C5—H5B	0.9600
C20B—H20C	0.9700	C5—H5C	0.9600
C20B—H20D	0.9700	N7—HN7B	0.86 (3)
C16B—N8	1.445 (6)	N7—HN7A	0.83 (3)
C16B—C17B	1.498 (12)	C6—C7	1.483 (3)
C16B—H16C	0.9700	C6—H6A	0.9700
C16B—H16D	0.9700	C6—H6B	0.9700
C19B—C18	1.580 (9)	C10—C9	1.490 (4)
C19B—H19C	0.9700	C10—H10A	0.9700
C19B—H19D	0.9700	C10—H10B	0.9700
C17B—C18	1.553 (7)	C15—H15A	0.9600
C17B—H17C	0.9700	C15—H15B	0.9600
C17B—H17D	0.9700	C15—H15C	0.9600
N2—C1	1.342 (2)	C7—C8	1.524 (4)
N2—C2	1.344 (2)	C7—H7A	0.9700
N1—C4	1.344 (2)	C7—H7B	0.9700
N1—C1	1.345 (2)	C18—H18A	0.9700
N6—C11	1.331 (3)	C18—H18B	0.9700
N6—C12	1.342 (2)	C9—C8	1.514 (4)
C1—N3	1.343 (2)	C9—H9A	0.9700
N5—C11	1.337 (3)	C9—H9B	0.9700
N5—C14	1.351 (2)	C8—H8A	0.9700
N8—C12	1.358 (3)	C8—H8B	0.9700
			
C19A—C20A—N8	110.5 (7)	C4—C3—H3	120.9
C19A—C20A—H20A	109.6	C2—C3—H3	120.9
N8—C20A—H20A	109.6	N2—C2—N4	117.42 (17)
C19A—C20A—H20B	109.6	N2—C2—C3	120.31 (17)
N8—C20A—H20B	109.5	N4—C2—C3	122.24 (18)
H20A—C20A—H20B	108.1	N5—C14—C13	122.76 (19)
C17A—C16A—N8	108.1 (7)	N5—C14—C15	116.32 (18)
C17A—C16A—H16A	110.1	C13—C14—C15	120.91 (19)
N8—C16A—H16A	110.1	N1—C4—C3	122.61 (18)
C17A—C16A—H16B	110.1	N1—C4—C5	115.69 (17)
N8—C16A—H16B	110.1	C3—C4—C5	121.69 (18)
H16A—C16A—H16B	108.4	N6—C11—N5	127.62 (18)
C20A—C19A—C18	107.5 (7)	N6—C11—N7	116.2 (2)
C20A—C19A—H19A	110.2	N5—C11—N7	116.2 (2)
C18—C19A—H19A	110.2	C2—N4—C10	122.80 (19)
C20A—C19A—H19B	110.2	C2—N4—C6	122.29 (17)
C18—C19A—H19B	110.2	C10—N4—C6	112.38 (17)
H19A—C19A—H19B	108.5	C4—C5—H5A	109.5
C16A—C17A—C18	108.0 (6)	C4—C5—H5B	109.5
C16A—C17A—H17A	110.1	H5A—C5—H5B	109.5
C18—C17A—H17A	110.1	C4—C5—H5C	109.5
C16A—C17A—H17B	110.1	H5A—C5—H5C	109.5
C18—C17A—H17B	110.1	H5B—C5—H5C	109.5
H17A—C17A—H17B	108.4	C11—N7—HN7B	121.5 (18)
N8—C20B—C19B	107.4 (7)	C11—N7—HN7A	117.4 (19)
N8—C20B—H20C	110.2	HN7B—N7—HN7A	121 (3)
C19B—C20B—H20C	110.2	N4—C6—C7	110.8 (2)
N8—C20B—H20D	110.2	N4—C6—H6A	109.5
C19B—C20B—H20D	110.2	C7—C6—H6A	109.5
H20C—C20B—H20D	108.5	N4—C6—H6B	109.5
N8—C16B—C17B	108.6 (7)	C7—C6—H6B	109.5
N8—C16B—H16C	110.0	H6A—C6—H6B	108.1
C17B—C16B—H16C	110.0	N4—C10—C9	110.1 (2)
N8—C16B—H16D	110.0	N4—C10—H10A	109.6
C17B—C16B—H16D	110.0	C9—C10—H10A	109.6
H16C—C16B—H16D	108.3	N4—C10—H10B	109.6
C20B—C19B—C18	110.0 (6)	C9—C10—H10B	109.6
C20B—C19B—H19C	109.7	H10A—C10—H10B	108.2
C18—C19B—H19C	109.7	C14—C15—H15A	109.5
C20B—C19B—H19D	109.7	C14—C15—H15B	109.5
C18—C19B—H19D	109.7	H15A—C15—H15B	109.5
H19C—C19B—H19D	108.2	C14—C15—H15C	109.5
C16B—C17B—C18	110.2 (6)	H15A—C15—H15C	109.5
C16B—C17B—H17C	109.6	H15B—C15—H15C	109.5
C18—C17B—H17C	109.6	C6—C7—C8	112.2 (2)
C16B—C17B—H17D	109.6	C6—C7—H7A	109.2
C18—C17B—H17D	109.6	C8—C7—H7A	109.2
H17C—C17B—H17D	108.1	C6—C7—H7B	109.2
C1—N2—C2	116.66 (16)	C8—C7—H7B	109.2
C4—N1—C1	115.39 (15)	H7A—C7—H7B	107.9
C11—N6—C12	116.79 (17)	C19A—C18—C17A	110.8 (5)
N2—C1—N3	117.06 (17)	C19A—C18—C17B	115.2 (4)
N2—C1—N1	126.63 (16)	C17A—C18—C19B	116.0 (4)
N3—C1—N1	116.30 (16)	C17B—C18—C19B	104.4 (6)
C11—N5—C14	114.52 (17)	C19A—C18—H18A	109.5
C12—N8—C16B	116.9 (3)	C17A—C18—H18A	109.5
C12—N8—C20B	117.6 (3)	C19A—C18—H18B	109.5
C16B—N8—C20B	115.1 (4)	C17A—C18—H18B	109.5
C12—N8—C20A	125.1 (3)	C17B—C18—H18B	128.8
C16B—N8—C20A	117.9 (4)	C19B—C18—H18B	126.7
C12—N8—C16A	122.9 (3)	H18A—C18—H18B	108.1
C20B—N8—C16A	119.3 (4)	C10—C9—C8	110.9 (2)
C20A—N8—C16A	106.9 (5)	C10—C9—H9A	109.5
C1—N3—HN3B	117.9 (14)	C8—C9—H9A	109.5
C1—N3—HN3A	123.4 (13)	C10—C9—H9B	109.5
HN3B—N3—HN3A	118 (2)	C8—C9—H9B	109.5
C14—C13—C12	118.35 (18)	H9A—C9—H9B	108.0
C14—C13—H13	120.8	C9—C8—C7	111.2 (2)
C12—C13—H13	120.8	C9—C8—H8A	109.4
N6—C12—N8	117.23 (18)	C7—C8—H8A	109.4
N6—C12—C13	119.96 (18)	C9—C8—H8B	109.4
N8—C12—C13	122.81 (18)	C7—C8—H8B	109.4
C4—C3—C2	118.24 (18)	H8A—C8—H8B	108.0
			
N8—C20A—C19A—C18	−61.6 (12)	C4—C3—C2—N2	2.5 (3)
N8—C16A—C17A—C18	62.1 (10)	C4—C3—C2—N4	−175.7 (2)
N8—C20B—C19B—C18	59.8 (11)	C11—N5—C14—C13	0.5 (3)
N8—C16B—C17B—C18	−60.5 (12)	C11—N5—C14—C15	179.23 (19)
C2—N2—C1—N3	−178.88 (17)	C12—C13—C14—N5	−0.4 (3)
C2—N2—C1—N1	2.3 (3)	C12—C13—C14—C15	−179.1 (2)
C4—N1—C1—N2	1.2 (3)	C1—N1—C4—C3	−2.9 (3)
C4—N1—C1—N3	−177.69 (17)	C1—N1—C4—C5	176.35 (18)
C17B—C16B—N8—C12	−156.0 (6)	C2—C3—C4—N1	1.2 (3)
C17B—C16B—N8—C20B	59.8 (12)	C2—C3—C4—C5	−178.0 (2)
C17B—C16B—N8—C20A	26.6 (13)	C12—N6—C11—N5	−0.9 (3)
C17B—C16B—N8—C16A	−45.9 (10)	C12—N6—C11—N7	178.89 (18)
C19B—C20B—N8—C12	156.4 (6)	C14—N5—C11—N6	0.3 (3)
C19B—C20B—N8—C16B	−59.6 (12)	C14—N5—C11—N7	−179.58 (19)
C19B—C20B—N8—C20A	43.2 (8)	N2—C2—N4—C10	171.1 (2)
C19B—C20B—N8—C16A	−28.6 (12)	C3—C2—N4—C10	−10.7 (3)
C19A—C20A—N8—C12	−141.8 (6)	N2—C2—N4—C6	10.5 (3)
C19A—C20A—N8—C16B	35.4 (14)	C3—C2—N4—C6	−171.3 (2)
C19A—C20A—N8—C20B	−56.9 (9)	C2—N4—C6—C7	103.3 (2)
C19A—C20A—N8—C16A	63.1 (12)	C10—N4—C6—C7	−59.1 (3)
C17A—C16A—N8—C12	141.3 (5)	C2—N4—C10—C9	−101.0 (3)
C17A—C16A—N8—C16B	55.4 (10)	C6—N4—C10—C9	61.3 (3)
C17A—C16A—N8—C20B	−33.4 (11)	N4—C6—C7—C8	52.6 (3)
C17A—C16A—N8—C20A	−62.9 (10)	C20A—C19A—C18—C17A	60.3 (12)
C11—N6—C12—N8	−178.51 (18)	C20A—C19A—C18—C17B	27.0 (14)
C11—N6—C12—C13	0.9 (3)	C20A—C19A—C18—C19B	−46.2 (9)
C16B—N8—C12—N6	25.3 (5)	C16A—C17A—C18—C19A	−61.9 (12)
C20B—N8—C12—N6	168.6 (4)	C16A—C17A—C18—C17B	42.7 (7)
C20A—N8—C12—N6	−157.5 (5)	C16A—C17A—C18—C19B	−31.3 (13)
C16A—N8—C12—N6	−6.2 (5)	C16B—C17B—C18—C19A	33.2 (14)
C16B—N8—C12—C13	−154.1 (5)	C16B—C17B—C18—C17A	−55.6 (8)
C20B—N8—C12—C13	−10.8 (5)	C16B—C17B—C18—C19B	61.3 (12)
C20A—N8—C12—C13	23.1 (6)	C20B—C19B—C18—C19A	54.9 (9)
C16A—N8—C12—C13	174.4 (4)	C20B—C19B—C18—C17A	−31.0 (13)
C14—C13—C12—N6	−0.3 (3)	C20B—C19B—C18—C17B	−61.6 (12)
C14—C13—C12—N8	179.10 (19)	N4—C10—C9—C8	−56.8 (3)
C1—N2—C2—N4	174.23 (18)	C10—C9—C8—C7	51.3 (3)
C1—N2—C2—C3	−4.0 (3)	C6—C7—C8—C9	−49.5 (3)

### Hydrogen-bond geometry (Å, º)

**Table:**

*D*—H···*A*	*D*—H	H···*A*	*D*···*A*	*D*—H···*A*
N3—H*N*3*B*···N1^i^	0.86 (2)	2.19 (2)	3.043 (2)	173.3 (19)
N3—H*N*3*A*···N5	0.90 (2)	2.34 (2)	3.210 (2)	162.1 (17)

Symmetry code: (i) −*x*+1, −*y*+2, −*z*.
